# Assessment of eight nucleic acid amplification technologies for potential use to detect infectious agents in low-resource settings

**DOI:** 10.1371/journal.pone.0215756

**Published:** 2019-04-22

**Authors:** Jason L. Cantera, Heather White, Maureen H. Diaz, Shivani G. Beall, Jonas M. Winchell, Lorraine Lillis, Michael Kalnoky, James Gallarda, David S. Boyle

**Affiliations:** 1 PATH, Seattle, Washington, United States of America; 2 Centers for Disease Control and Prevention, Division of Bacterial Diseases, Respiratory Diseases Branch, Atlanta, Georgia, United States of America; 3 Bill and Melinda Gates Foundation, Seattle, Washington, United States of America; University of Helsinki, FINLAND

## Abstract

Nucleic acid amplification technologies (NAATs) are high-performance tools for rapidly and accurately detecting infectious agents. They are widely used in high-income countries to diagnose disease and improve patient care. The complexities associated with test methods, reagents, equipment, quality control and assurance require dedicated laboratories with trained staff, which can exclude their use in low-resource and decentralized healthcare settings. For certain diseases, fully integrated NAAT devices and assays are available for use in environmentally-controlled clinics or emergency rooms where relatively untrained staff can perform testing. However, decentralized settings in many low- and middle-income countries with large burdens of infectious disease are challenged by extreme environments, poor infrastructure, few trained staff and limited financial resources. Therefore, there is an urgent need for low-cost, integrated NAAT tools specifically designed for use in low-resource settings (LRS). Two essential components of integrated NAAT tools are: 1) efficient nucleic acid extraction technologies for diverse and complex sample types; and 2) robust and sensitive nucleic acid amplification and detection technologies. In prior work we reported the performance and workflow capacity for the nucleic acid extraction component. In the current study we evaluated performance of eight novel nucleic acid amplification and detection technologies from seven developers using blinded panels of RNA and/or DNA from three pathogens to assess both diagnostic accuracy and suitability as an essential component for low-cost NAAT in LRS. In this exercise, we noted significant differences in performance among these technologies and identified those most promising for potential further development.

## Introduction

The accurate and rapid diagnosis of infectious disease can lead to improved patient care, appropriate use of antimicrobial drugs, and ultimately greater control of communicable diseases within populations [1]. The advent of molecular tests to accurately identify pathogen DNA or RNA has revolutionized clinical diagnostic testing, and molecular testing is now routine in many settings. Implementation is enabled in high-income countries by centralized laboratories with appropriate infrastructure and controlled environments to house complex equipment and cold chain–dependent reagents. These facilities also have highly skilled staff to operate and maintain the equipment under quality assurance protocols. In countries with sufficient resources, a centralized model offers high-quality diagnostic testing to most of the population because logistic infrastructure enables efficient shipping of samples and communication of test results. However, low-income countries face greater challenges because the burden of infectious diseases is much higher and access to healthcare is limited due to inadequate resources and poor infrastructure [2].

In low- and middle-income countries (LMICs), some centralized laboratories perform complex molecular testing (e.g., HIV viral load measurement for treatment monitoring), but these facilities are often in urban settings. It is difficult for patients in rural or peri-urban regions to access these services, in part due to unreliable infrastructure and transportation options [3]. Therefore, offering diagnostic testing in lower-tier local facilities may improve quality of care and patient outcomes.

During the past decade, significant investments in engineering, software, and reagent formulations have resulted in the commercialization of stringent regulatory authorities- regulated nucleic acid amplification technology (NAAT) products in high income countries [4]. These in vitro diagnostic (IVD) products offer performance similar to that of dedicated laboratory-developed tests (LDT). In addition, with the integration and automation of nucleic acid extraction and purification, amplification, detection, and data analysis, these integrated platforms allow minimally skilled workers to produce rapid and reliable test results. Some of these platforms are now used for the diagnosis of globally significant infectious diseases, such as HIV and tuberculosis (TB) [5–8].The GeneXpert instrument platform (Cepheid Inc., Sunnyvale, CA, USA), together with associated Xpert MTB/RIF assay cartridge, is a prominent integrated NAAT platform in global health, being used in more than 96 countries to diagnose rifampicin-resistant TB [5,9]. The Xpert test menu has been expanded to include assays for HIV infection and HIV viral load [10,11]. Alere Inc. (now Abbott Molecular) and Roche Diagnostics have also developed integrated platforms, the m-PIMA (previously the Alere q) and cobas Liat instruments, respectively, for early infant detection of HIV [8], viral load [12,13], and MDR TB [7].

Although the development of these platforms is encouraging, challenges exist for their effective and sustained use in LRS. The GeneXpert platform has been affected by elevated ambient temperatures and dust, which largely limits its location to environmentally-controlled reference and intermediate-level laboratories [2,9]. Cepheid is developing the GeneXpert Omni and Edge platforms, instruments intended to be more environmentally robust [14]. The current Roche Liat assays require cold chain storage, which limits their use in environmentally challenging settings [13]. The m-PIMA and associated HIV-1/2 and MDR TB tests were designed for use in austere settings, and early performance data from hospital settings are encouraging [15–17]. The menu of tests for other infectious diseases on the integrated NAAT platforms are primarily for high income country markets, including hospital acquired infections, respiratory pathogens, diarrheal diseases, and gonorrhea/chlamydia. Such assays are also of value in LMICs, but other diseases that have significant associated mortalities if left untreated (e.g. malaria, meningitis, pneumonia, or typhoid) are not included in integrated NAAT test menus. The emergence of Zika virus led to significant NAAT development efforts with at least fourteen groups having received emergency use authorization (EUA) from the US Food and Drug Administration to meet the needs of the US market but these are for use in open systems or with larger automated platforms (e.g. the Roche cobas 6800 or 8800 Systems) [18]. By comparison, the EUA clearances to rapidly develop diagnostic assays for Ebola virus include three fully integrated assays hosted on the BioFire (bioMérieux, France, GeneXpert (Cepheid, USA) and Idylla platforms (Biocartis and Janssen Diagnostics, both Belgium); this is likely due to the very significant biohazard risks associated with handling Ebola infected specimens and the lack of appropriate laboratory infrastructure in Ebola affected regions [19].

Cost is another critical issue—the global donor supported cost of US$9.98 per GeneXpert MTB/RIF cartridge is seen as unaffordable for many LMIC TB programs [9]. The MTB/RIF cartridge for use with the Omni is projected to increase to $11.40 due to adding a near field communication chip to the test cartridge [20]. The HIV-1 assays from Cepheid and Abbott are estimated to cost $17.95 and $25.00, respectively [20,21]. By contrast, a consensus meeting of TB experts suggested that the cost for a replacement test for sputum smear microscopy should be in the range of $4 to $6 [22]. Therefore, while it is exciting that new diagnostic tools are becoming available to improve clinical care in austere environments, the cost per test is a key barrier to uptake and sustained use [22].

The goal of this project was to investigate inexpensive nucleic acid amplification and detection technologies that can potentially be integrated into “sample to answer” NAAT platforms that are suitable for low-resource settings (LRS). In a companion paper we compared the performance and workflow characteristics of six nucleic acid extraction technologies for suitability in LRS [23]. These technologies are to be designed for primary use by minimally skilled operators, and so will require limited training and as few manual steps as possible. In the present study, we assessed the sensitivity, specificity, time to result, and complexity of eight novel nucleic acid amplification and detection technologies for potential use in LRS. Based on these criteria, we were able to identify technologies that have the potential to meet the required specifications for use in LRS [19].

## Materials and methods

### Identification of technology developers

An extensive search of peer-reviewed literature, published technical reports and white papers, commercial literature, company websites, conference proceedings, and letters of interest to a donor–the Bill & Melinda Gates Foundation (BMGF) was conducted to identify multiple technology developers with innovative nucleic acid (NA) amplification and detection methods that have the potential for use in challenging environments by minimally skilled users, or capable of being engineered to require minimum hands on time by the user Seven developers agreed to participate in this study based upon the current maturity of their technology and the potential to meet the cost goal of approximately $3. The institutions were comprised of a variety of companies or university groups; Akonni Biosystems, Inc. (Frederick, MD, USA), Alveo Technologies (Alameda, CA, USA), Integrated Nano-technologies (Henrietta, NY, USA), Friz Biochem (Neuried, Germany), Tangen Biosciences, (Branford, CT, USA), Two Pore Guys (Santa Cruz, CA, USA) and University of Washington (Seattle, WA, USA). All of these groups are small in size and most are relatively new companies. Although these institutions had no role in designing the study, the authors agreed that the identities of each would be coded for this report if the groups wished to be anonymous. While there is a lot of studies reflecting the performance of different commercial assays or the comparison of laboratory designed assays versus a gold standard test, this study is relatively unique in that not only was the performance of multiple amplification assays assessed but also the detection platforms unique to these companies. The institutions have developed NA amplification and detection methods and devices incorporating a variety of innovative technologies, including fluorescence, immunocapture, and electrochemical methods [24]. The operating platforms ranged from fully integrated devices to prototype systems that still require some development to remove multiple manual steps prior to and/or during processing. Each of the developers received blinded test panels containing microbial DNA and/or RNA derived from three pathogens to test in their facilities. Developer F offered two different amplification methods using either PCR or loop mediated amplification (LAMP).

### Materials for the construction of nucleic acid panels

We chose influenza A virus (a RNA-based pathogen), *Mycobacterium tuberculosis* (MTB), and *Salmonella enterica* serovar Typhimurium (both DNA-based pathogens) to prepare our test panels. Cultured influenza A virus (H3N1) supernatant was a gift from the Washington State Public Health Laboratories (Shoreline, WA, USA). DNA extracted from *M*. *tuberculosis* H37Rv strain Johannesburg was procured from the laboratory of Dr. B. Kana (University of Witwatersrand, Johannesburg, South Africa). *S*. Typhimurium LT2, a gift from Dr. S. Meschke (University of Washington, Seattle, WA, USA), was cultured in tryptic soy broth (Sigma-Aldrich, St. Louis, MO, USA) with overnight incubation at 37°C with shaking. The cells were pelleted by centrifugation, washed once with phosphate-buffered saline (PBS), resuspended in 5 mL PBS, and stored at 4°C until DNA extraction. With the exception of MTB, whose DNA was received as already purified, the extraction of the cultured bacterial and viral nucleic acids was performed using kits from Qiagen (Hilden, Germany) following the kit instructions. Influenza A samples were processed using the QIAamp Viral RNA Mini Kit. The input sample was 140 μL volume of culture supernatant, with RNA eluted in a final volume of 60 μL. DNA extraction from *S*. Typhimurium stocks used the Qiagen DNeasy kit. A 500 μL volume of washed cells was processed per column and eluted in a final volume of 200 μL. All nucleic acid extracts were stored at –20°C until use.

### Real-time RT PCR quality control of extracted nucleic acids

Nucleic acid extracts were qualified prior to construction of blinded panels via real-time reverse transcription polymerase chain reaction (RT PCR) for influenza A, or PCR for the DNA-based pathogens. Oligonucleotide primers and probes were purchased from Integrated DNA Technologies, Inc. (Coralville, IA, USA). The core amplification reagent used in all real-time assays was qScript XLT 1-Step RT-qPCR ToughMix (Quanta Bio, Gaithersburg, MD, USA), regardless whether a RT step was required. The real time RT PCR assays used in the PATH laboratory for each pathogen NA were as follows: for influenza A, the universal influenza A RT PCR assay [25]; for MTB, an assay that targets the insertion sequence IS*6110* [26]; and for *S*. Typhimurium, an assay targeting the *hns* gene was used [27]. All reactions were prepared as 20 μL volumes to which 5 μL of nucleic acid extracts were added to make a final reaction volume of 25 μL. Amplification and detection in real time was carried out using an Mx3005P qPCR system (Agilent Technologies, Santa Clara, CA, USA) using the FAM detector channel during the 60°C annealing stage. The Centers for Disease Control and Prevention (CDC) PCR protocols were different for MTB and Salmonella [28,29], but the core reagents were the same as described earlier. Each reaction used 5 μL of sample in a final reaction volume of 25 μL. The amplification protocol for reverse transcription used an incubation step of 45°C for 10 minutes followed by a step of 95°C for 10 min. PCR amplification then entailed 45 cycles of 30 seconds at 90°C and 1 minute at 60°C. Fluorescence was measured in real time using the FAM channel during the 60°C stage. The CDC laboratory used the 7500 Fast Dx system (Applied Biosystems, Foster City, CA, USA).

### Construction of NA test panels

Three test panels representing MTB (panel MTB), influenza A (panel INF) and *S*. Typhimurium (panel SAL) were made. Each panel consisted of six members, five containing different amounts of target NAs and one unspiked for use as a specificity control. The diluent used was 10 mM Trizma base (pH 8.0; Sigma-Aldrich, St. Louis, MO, USA) spiked with 5 μg/mL of human DNA (Promega Corp, Madison, WI, USA) to mimic expected human DNA co-extraction from clinical samples, and to serve as an internal amplification control if elected by the developers.

Because the intent of the study was to assess the performance of NA amplification and detection methods, sensitivity of the candidate technologies was the primary performance metric for comparison. Therefore, the panel designs were biased toward samples that had limited target NA for amplification to identify assays that were capable of detecting very low levels of target. Initially, we diluted the NA extracts until each stock solution had a C_t_ value of 19 (± 0.5). Assuming the PCR assays had a theoretical efficiency of 100%, ten-fold dilutions of each NA extract should result in an increase in the C_t_ value by 3.3 cycles per log dilution. Ten-fold dilutions of the stocks create a series of samples where the C_t_ values derived from five such tenfold dilutions are calculated to be approximately 22.5, 25.8, 29.1, 32.4, and 35.7.

To prevent cross-contamination, the negative control stocks of 10 mM Tris with 5 μg/mL human DNA were aliquoted in multiple 10 μL aliquots before the target NAs were prepared. The five consecutive ten-fold dilutions were then prepared from the DNA or RNA stock solutions each having an initial C_t_ value of ~19.0, wherein 100 μL of material was added to 900 μL of Tris buffer (containing human DNA as background) at each dilution step. Mixing was critical in this process, especially for the DNA-based targets at the lowest dilutions, to ensure even distribution of the genomic DNAs. After a NA solution was transferred to its diluent, each sample was then vortexed six times in 10-second intervals. The tube was then briefly centrifuged prior to making the next dilution. Once each panel’s dilution series was completed, multiple 10 μL aliquots were immediately stored at –20°C. Subsets of these NA samples were then tested for quality control by real-time RT PCR to confirm that each dilution contained an appropriate amount of challenge material. Samples from the three panels were shipped on dry ice overnight to the CDC to independently replicate the initial test data and confirm the composition of the panels.

### Shipping and use of the panels

To ensure developers had the opportunity to optimize the performance of their assays before the blinded panels were shipped, each group received aliquots of the purified NA extracts for each of the three pathogens used in this study. After this phase, the participating developers then received six replicates for each of the five titered levels across the three pathogen panels (total 90 panel members). Each panel member was blinded via four-digit numbering and the sets shipped on dry ice via an overnight courier with confirmation of integrity of cold chain upon receipt. To ensure that each developer processed the same relative amount of sample, they were asked to use the equivalent of 2.5 μL from each blinded sample for their respective NA amplification/detection technologies. For example, if an assay requires a 10 μL sample input, then 10 μL test sample would first be diluted with 30 μL nuclease-free water to a 40 μL final volume, mixed, and then 10 μL of the diluted sample would be used in the assay (equivalent to 2.5 μL of sample and 7.5 μL of nuclease free water). This requirement ensured that developers used the same amount of input material regardless of the volume required by their assays (which we assumed would be variable). This approach allowed for a direct comparison of sensitivity between the methods as dictated by the volumes required for each developer. The data were sent to PATH upon completion of testing. The technologies were ranked based on assay performance; however, assay run time and number of user steps were also evaluated for each technology. When all testing and analysis was complete, the data were unblinded to each developer.

## Results

### Panel quality confirmation in-house and by a reference laboratory

After the three test panels were constructed and prepared, PATH screened all samples for each dilution in triplicate RT PCR reactions (Table 1). Each sample tested consistently gave the anticipated result, although the C_t_ values from the replicate tests from each tenfold dilution varied slightly (typically +/- 0.3 C_t_) from the predicted values, which was likely due to potential uneven distribution of NAs during mixing and less than 100% efficiency of the PCR assays [30]. Because the NAs at all dilutions were consistently detected, the panels were deemed as suitable to assess and verify the performance of the developer NAATs. PATH shipped three blinded aliquots of each sample from all panels to the CDC laboratory for independent confirmation of the panels test results via the same or similar real-time PCR methods. All samples from the MTB, INF, and SAL panels were concordant for negative and positive samples (Table 1). The mean C_t_ values generated from either laboratory showed minimal variability for each panel (generally within 1–2 C_t_ values). The CDC used different assays and a different real-time PCR platform to detect the MTB and Salmonella DNAs, which are likely reasons for some variation in C_t_ values. Nonetheless, each sample tested at the CDC provided the expected semi-quantitative result when compared to the original PATH laboratory data. With this confirmatory analysis, the blinded panels were subsequently shared with the participating developers for their independent analyses.

**Table 1:** A comparison of PATH and CDC real-time PCR data with the test panel scores from each developer. The PATH and CDC datasets are shown as the mean C_t_ from three reactions per sample dilution. The developer datasets are depicted as the number of correct results scored for each sample (maximum 5) as compared to the PATH test data. C_t_, mean cycle threshold; A–G denotes the different developers; F1, developer F used PCR amplification; F2, developer F used loop-mediated isothermal amplification; NT, not tested; N/A not applicable; CI, confidence interval.

Panel	Sample	PATH (Ct)	CDC (Ct)	Developer (correct test scores - N = 5 replicate samples)							
				A	B	C	D	E	F1	F2	G
INF	1	21.9	24.1	5	5	5	5	NT	5	5	1
	2	25.5	27.5	5	5	5	5	NT	5	5	0
	3	28.8	30.6	5	5	5	4	NT	5	5	0
	4	32.1	34.2	5	4	5	1	NT	5	5	0
	5	35.6	37.4	3	0	5	0	NT	5	4	0
	6^a^	Neg.	Neg.	5	5	5	5	NT	5	5	5
	**Rank**			**4**	**5**	**1**	**6**	**N/A**	**1**	**3**	**7**
	Sensitivity			0.92	0.76	1.0	0.6	NT	1.0	0.96	0.07
	CI (95%)			(0.74,0.99)	(0.55,0.91.0)	(0.86,1.0)	(0.39,0.79)	NT	(0.86,1.0.0)	(0.8,1.0)	(0.0,0.32)
	Specificity			1.0	1.0	1.0	1.0	NT	1.0	1.0	1.0
	CI (95%)			(0.48,1.0)	(0.48,1.0)	(0.48,1.0)	(0.48,1.0)	NT	(0.48,1.0)	(0.48,1.0)	(0.16,1.0)
MTB	7	22.7	24.4	5	5	5	4	5	5	5	5
	8	26	28.1	5	5	5	4	5	5	5	4
	9	29.5	31.7	5	5	5	3	4	5	5	1
	10	32.7	34.8	5	1	5	0	0	5	5	2
	11	35.9	38	1	2	5	0	1	4	1	2
	12^a^	Neg.	Neg.	5	5	5	5	5	5	5	2^b^
	**Rank**			**3**	**5**	**1**	**7**	**6**	**2**	**3**	**7**
	Sensitivity			0.84	0.72	1	0.48	0.6	0.96	0.84	0.58
	CI (95%)			(0.64,0.95)	(0.51,0.88)	(0.86,1.0)	(0.27,0.69)	(0.39,0.79)	(0.8,1.0)	(0.64,0.95)	(0.37,0.78)
	Specificity			1	1	1	1	1	1	1	0.6
	CI (95%)			(0.48,1.0)	(0.48,1.0)	(0.48,1.0)	(0.48,1.0)	(0.48,1.0)	(0.48,1.0)	(0.48,1.0)	(0.15,0.95)
SAL	13	22.2	21	5	5	5	4	5	5	5	5
	14	26.1	24.3	5	5	5	3	5	5	5	4
	15	29.5	27.8	5	4	5	3	4	5	3	2
	16	33	31	3	1	5	4	0	5	4	1
	17	37.4	34.5	0	0	5	0	0	5	1	0
	18^a^	Neg.	Neg.	5	5	5	5	5	5	5	3^b^
	**Rank**			**3**	**5**	**1**	**6**	**6**	**1**	**3**	**8**
	Sensitivity			0.72	0.6	1.0	0.56	0.56	1.0	0.72	0.48
	CI (95%)			(0.51,0.88)	(0.39,0.79)	(0.86,1.0)	(0.35,0.76)	(0.35,0.76)	(0.86,1.0)	(0.51,0.88)	(0.28,0.69)
	Specificity			1.0	1.0	1.0	1.0	1.0	1.0	1.0	0.4
	CI (95%)			(0.48,1.0)	(0.48,1.0)	(0.48,1.0)	(0.48,1.0)	(0.48,1.0)	(0.48,1.0)	(0.48,1.0)	(0.05,0.85)
	**Overall Rank**			**3=**	**5**	**1**	**6**	**7**	**2**	**3=**	**8**

^a^ Negative sample

^b^ some negative samples were incorrectly scored as positive

INF, influenza A; MTB, *M*. *tuberculosis*; SAL, *S*. Typhimurium.

### Test data from the participating developers

**Fig 1:**
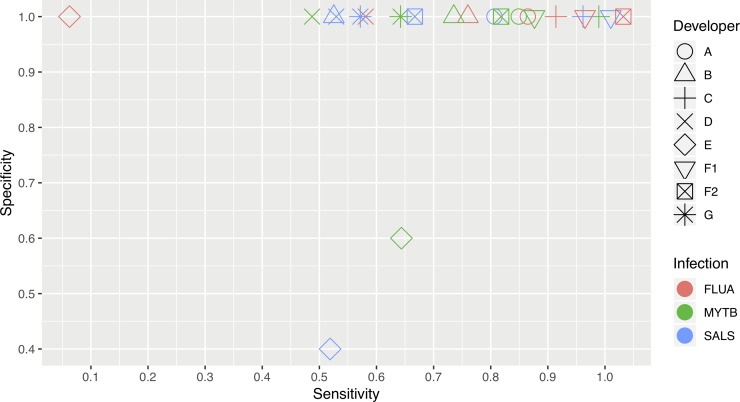
**Comparison of the sensitivity and specificity observed for each developer’s (A–G) assays for the three microbial targets.** Where developers’ data points are overlapping, the icons are slightly dispersed for greater clarity. INF, influenza A; MTB, *M*. *tuberculosis*; SAL, *S*. Typhimurium.

Each of the seven developers completed panel testing within the allotted time and sent datasets to PATH. Prior to testing, developers were asked to use the normalized equivalent of 2.5 μL from each blinded sample, regardless of the volume specified for amplification on their platform. This directive ensured that each developer applied the same amount of RNA or DNA, allowing for a direct comparison across technologies. As the goal of the evaluation was to determine the performance of each technology’s ability to detect the target at different concentrations, each dataset was evaluated based on correctly identifying the presence or absence of the various targets. Upon receipt of all datasets, the panel compositions were unblinded to each developer (Table 1). Developer E did not have a functional assay for influenza A and did not provide data for this panel. The sensitivities and specificities for each developer were graphically plotted for each of the three test agents (Fig 1). While most developers achieved high specificity, there was considerable variation observed in sensitivity. Annotated and complete PCR data sets and developer test results are publicly available (https://dataverse.harvard.edu/dataverse/SSB).

With the INF panel, each of the developers correctly identified the negative samples, as well as the higher-spiked samples (samples 1–3), except for developer E. Developer G incorrectly identified 3/5 and 2/5 negative samples as positive for MTB and AL, respectively; these were the only false positive results obtained by any developer for any panel. Developers C and F (when F used an RT PCR–based assay–F1) correctly identified all of the influenza A-positive samples. Developer A failed to detect one replicate from sample 5, the lowest RNA concentration, and developer F also failed to detect one replicate from sample 5 when using RT LAMP. Developers B and D failed to detect RNA within all replicates containing the lowest concentration of this nucleic acid (sample 5). Developers D also failed to detect influenza A RNA in all replicates of samples 3 and 4, and developer G only detected influenza A RNA in one replicate of the highest concentration sample.

All developers correctly identified the MTB-negative samples, except for developer G. Developer C correctly identified MTB DNA in all spiked replicates, and developer F missed only one of five replicates at the lower range (sample 11) when using PCR amplification (Table 1). The other developers demonstrated limited detection of replicates for samples 10 and 11, those with the lowest levels of DNA; D and G did not detect any MTB DNA in sample 10. The lowest levels of performance were observed with Developers D and E which scored the lowest sensitivities for MTB DNA and developer G scoring lowest for specificity for MTB.

For the SAL panel, Developers C and F (when using PCR amplification, F1) correctly scored all positive sample replicates, including the lowest spiked member, sample 17. Interestingly, the low level of SAL DNA in sample 17 was not detected by the other developers, and the LAMP assay from developer F (F2) produced only one positive replicate (1/5) for this sample. The moderate amounts of SAL DNA in samples 15 and 16 demonstrated improved rates of positivity for most developers. The specificity of tests for the SAL panel was typically excellent (5/5), with all but one developer correctly identifying the negative panels. Developer G had two false positive results.

When the test scoring data from the three panels were pooled, developer C had the best performance based on correctly identifying every positive or negative replicate member in each of the three panels. The performance of developer F was similar, with only a single false-negative result at the lowest concentration of influenza A when using RT PCR–based detection (F1, Table 1). The pooled performance scores for other developers were more variable, with developers A and F2 (using the LAMP method) tying for third-best performance. Developer G had the poorest performance (excluding developer E with no submitted results).

Despite efforts to optimize assays before testing, developer E encountered persistent inhibition with the human genomic DNA in the panel samples for both the influenza A and salmonella assays. To limit the negative effects of the human DNA, developer E elected to use only 0.5 μL of each sample as opposed to the recommended 2.5 μL, effectively using only 20% of the test material as compared with the other developers. This may explain the limited sensitivity of developer E’s assays, especially at the lower concentration ranges.

**Fig 2:**
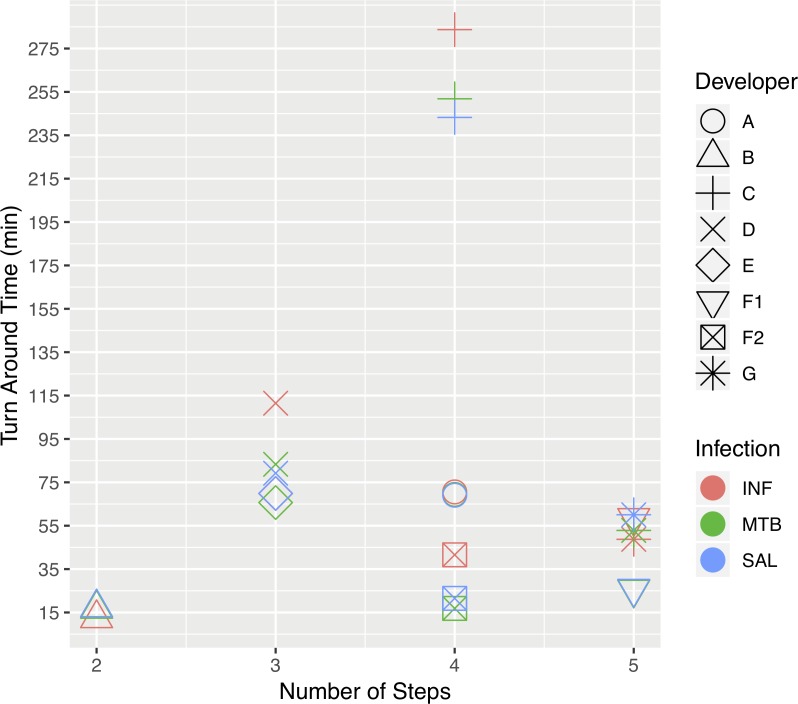
A comparison of the number of steps and the turnaround time for each product. Operational data were provided by each developer for amplification and detection of both RNA- and DNA-based targets. Where developers’ data points are overlapping, icons are slightly dispersed for greater clarity. A–G represent the seven developers; developer E did not perform RNA testing; F1, developer F used RT PCR; F2, developer F used LAMP; INF, influenza A; MTB, *M*. *tuberculosis*; SAL, *S*. Typhimurium.

In addition to assessing the analytical diagnostic accuracy, we investigated operational aspects of testing for each technology, whereby the number of steps required for each assay’s workflow was plotted against the overall turnaround time (TAT) to obtain a test result (Fig 2). The number of key steps listed by the developers was varied, with two to five operator steps required for assay preparation, amplification, and scoring the test result. However, there were more significant differences in TATs. Four assays took less than 1 hour, three required less than 2 hours, and the assay by developer C took almost 4.5 hours regardless of the nucleic acid tested (Fig 2).

The most rapid assays were the LAMP assay from developer F (F2) and the PCR assay from developer B; both methods had the same TAT irrespective of whether there was an RT step with subsequent DNA amplification (e.g., INF RNA) or not (e.g., MTB DNA). Isothermal methods can simultaneously employ RT and DNA amplification in the same incubation step because the RT and DNA polymerase use the same incubation temperature, reducing the time to amplify complementary DNA (cDNA) derived from RNA as opposed to traditional RT PCR methods [31,32]. RT PCR typically requires an initial reverse transcription step to generate cDNA before thermal cycling because the RT species used in molecular assays are denatured at elevated temperatures. Therefore, PCR cannot proceed until cDNAs are synthesized from the target RNA [33]. Despite this, developer B achieved detection of RNA in less than 1 hour using RT PCR. Assays from the next three developers (A, D, and G) required 1–2 hours. Developer C had the longest TAT of over 5 hours due to their methodology for interrogation of amplicons. They use a probe-based array that requires a hybridization stage from which to interrogate PCR amplicons. All developers used RT PCR with the exception of F2 and developer E, both of which used isothermal amplification methods.

The developers A, C, and F scored best overall in this exercise and are Friz Biochem, Akonni Biosystems and Two Pore Guys, respectively. In summary, the assays from Akonni Biosystems (C) were the most accurate but required a significantly longer TAT in comparison to the other developers. This was due to the Akonni test platform requiring a 3-hour DNA microarray hybridization step after amplification by asymmetric PCR [34,35]. The assays from Two Pore Guys (F) were next best in terms of sensitivity when using either PCR (F1) or LAMP (F2) and provided a very short TAT (20 and 35 minutes for LAMP and PCR, respectively). Friz (A) scored the same as F2 for test performance and had a moderate TAT (<1.5 hrs.) in terms of the assays tested.

## Discussion

The overarching intent of this work and its companion study was to identify suitable component technologies for the development of affordable and effective fully integrated NAAT devices to diagnose globally-relevant infectious diseases in LRS [36]. In this report, the key components focus on the timely and effective amplification and detection of pathogen nucleic acids. We worked with a series of innovative developers to explore their proprietary platforms and NA amplification assays for influenza A, MTB, and salmonella. The assays and platforms were challenged with blinded replicate series of dilution panels to assess their performance with an emphasis on sensitivity, a critical parameter for the success of any NAAT-based technology. Although the developers’ identities were coded to encourage participation in this head-to-head challenge, further details can be requested from each. The assays and devices screened in this study were in varying phases of the development pipeline, and any limited performance observed in this study may be related simply to early prototypes still in the development, as compared to mature devices in production.

The use of low concentrations of nucleic acids for testing was a key feature as it enabled insight into the limits of detection for each technology, and the study clearly identified the most sensitive technologies. An unexpected outcome was observed when adding human DNA as an optional internal control to qualify reagents and equipment performance. Although no developer chose to present data for this, the addition of human DNA highlighted one technology whose performance was adversely affected. A further point is that the addition of a small uniform amount of DNA does not necessarily mimic the actual dynamics of total nucleic acid recovered from clinical specimens due to the specimen type, heterogeneity and the method of nucleic acid extraction used. As it is expected that some human DNA will be present in most samples, ideally the technologies should be able to accommodate some degree of non-specific DNA/RNA from the host and in some instances commensal microflora (e.g. stool).

Interestingly, while there are many publications that advocate use of isothermal amplification methods in LRS based on rapid TAT, reduced power requirements, and simplicity of device design, only two isothermal methods were offered by developers in the current study [24]. The LAMP assays (F2) from Two Pore Guys performed reasonably well but the isothermal method from developer E demonstrated relatively poor performance. We suspect that device developers prefer to use PCR-based methods as a result of familiarity with the technique and experience with assay design in addition to a variety of technical reasons. The ubiquity of PCR means that many vendors offer high-quality core reagents with exceptional performance in terms of sensitivity, specificity (e.g. proof reading and hot start), fidelity, processivity, and multiplexing capability. These reagents are widely available with competitive prices given that intellectual property for PCR is now largely off-patent. By contrast, most isothermal technologies are still under patent, and the holders of these patents may have their own devices in development, offer only reagents, or lack an integrated platform with which to perform test reactions.

The detection modalities used by these tools included real-time and endpoint detection of amplification via fluorescence, optical imaging system of hapten labeling, immunochromatography, and electrochemistry. Of these methods, the fluorescence detection modalities gave the most rapid TAT, as seen by developers B and Two Pore Guys (both F1 and F2). The other methods were slower, giving results in 1 to 2 hours. The requirement to hybridize test reactions to an array for detection slowed the TAT for Akonni, but this also gave maximal sensitivity and specificity which were the primary performance attributes sought in this exercise. Akonni have worked to overcome the inherent complexity of microarray-based diagnostics for routine use by combining asymmetric PCR amplification, microarray hybridization and waste fluid retention to within a single microfluidic chamber. The detection of array bound Cy3 labelled PCR products is enabled via a low-cost, field-portable microarray imager that Akonni is developing [35].

Interestingly the detection modalities from Two Pore Guys and Friz use electrical-based detection of target amplicons. Such systems are of great interest in austere environments as the technical design is much simpler than optical detection systems [37]. There may be further advantages to lower cost of production with solid state devices. As their name suggests, the Two Pore Guys technology uses a silicon chip that separates two fluidic volumes with amplicons pulled through a 20 nm nanopore by voltage. The amplicons are first labelled with a sequence specific molecular payload (probe) that cause a predefined shift in the trans‐pore ionic charge as they are pulled through the pore [38]. Different sized probes enables multiplexed detection and the developers claim single molecule sensitivity. The Friz approach is different, measuring conduction via an integrated gold plated micro-electrode-array (MEA). Pathogen specific labelled probes are immobilized on the MEA and create a high electrochemical signal prior to amplification. The increasing presence of PCR amplicons promotes the displacement of the labelled probe from the test site, thus reducing electrochemical signal and indicating the amplification of specific target DNAs. If a target is not amplified then the high signal remains uniform as the probe remains bound to the MEA. While Two Pore Guys and Friz use PCR, any amplification method that creates a complementary DNA for competitive binding to the detector probe modalities may be used, (e.g. the LAMP format with F2).

We identified two technologies from Akonni and Two Pore Guys with nearly identical performance across all panels. These and other technologies such as Friz are also under review for potential further development, especially considering other key factors including manufacturing scale, access to core intellectual property, and ability to achieve an estimated COGs of approximately US$3 (targeting US$5 per test when combined with a suitable NA extraction method). Cost is a critical issue. Although several integrated NAATs with the potential for use in LRS currently exist, the cost per test is generally judged unaffordable for infectious disease diagnosis in most low- and middle-income countries. Together, this study and the companion assessment of nucleic acid extraction technologies have identified several promising candidate technologies for integration into low-cost integrated sample-to-answer platforms for infectious disease diagnosis. Further development will aim to integrate technologies and reach manufacturing scale at low cost with the potential to offer rapid and accurate diagnosis of the highest priority pathogens in LMICs.
